# P-905. Applying Encounter-level Risk-Adjustment for Standardized Antimicrobial Administration Ratios (SAAR): Is it meaningful?

**DOI:** 10.1093/ofid/ofaf695.1111

**Published:** 2026-01-11

**Authors:** Rebekah W Moehring, Elizabeth Dodds Ashley, Rachel Addison, Whitney Buckel, Sara E Cosgrove, Eili Klein, Carlos Santos, Michael J Smith, Emily S Spivak, William Trick, David J Weber, Congwen Zhao, Deverick J Anderson, Ben Goldstein, Michael E Yarrington

**Affiliations:** Duke University, Durham, NC; Duke Center for Antimicrobial Stewardship and Infection Prevention, Durham, NC; Duke Center for Antimicrobial Stewardship and Infection Prevention, Durham, NC; Intermountain Health, Salt Lake City, UT; Johns Hopkins School of Medicine, Baltimore, MD; Johns Hopkins School of Medicine, Baltimore, MD; Rush University Medical Center, Chicago, Illinois; Duke University, Durham, NC; University of Utah School of Medicine, Salt Lake City, Utah; Cook County Health and Rush University Medical Center, Chicago, IL; University of North Carolina, Chapel Hill, NC; Duke University, Durham, NC; Duke Center for Antimicrobial Stewardship and Infection Prevention, Durham, NC; Duke University, Durham, NC; Duke University Health System, Durham, North Carolina

## Abstract

**Background:**

Whether encounter-level risk-adjustment impacts antimicrobial stewardship program (ASP) assessments and perceptions of standardized antimicrobial administration ratio (SAAR) comparisons is unknown.

**Figure:**
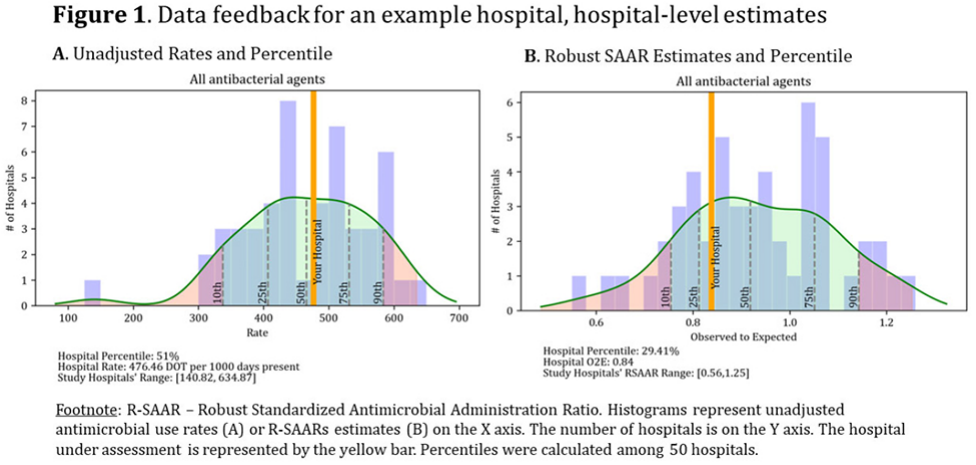

**Figure:**
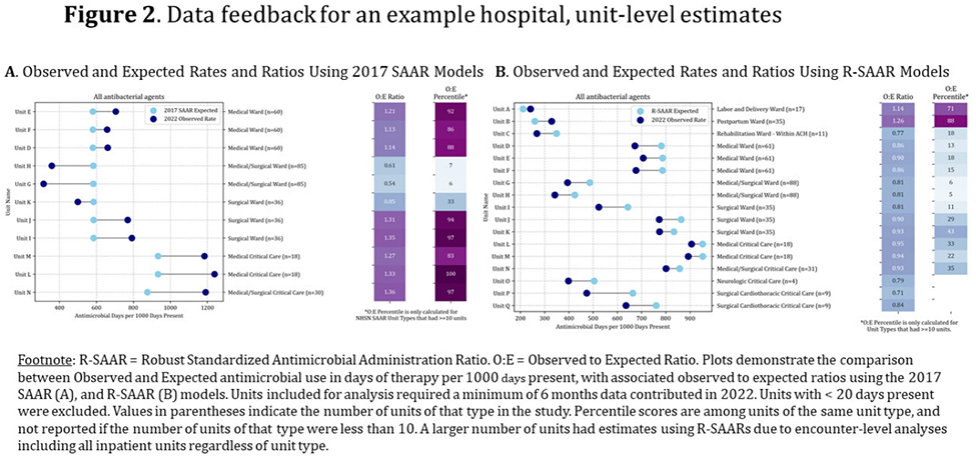

**Methods:**

This retrospective analysis included patient encounter-level data among 50 US hospitals participating in the CDC Epicenters collaborative, including 2022 antimicrobial use (AU), demographics, and diagnosis and procedure categories. We developed two-staged, xgboost machine learning models on 2020-2021 training sets for adult and pediatrics (aged 1-17) to produce expected days of therapy (DOT) for each NHSN antimicrobial group and each 2022 encounter. Observed and expected DOT were aggregated to create ratios (O:E), summarized as “robust” SAARs (R-SAARs) for hospital and unit. Methods and interpretation guidance were provided to ASPs in documents and recorded videos. Two hospital-specific reports were delivered sequentially over a 3-month period in 2024. Part 1 included unadjusted hospital-level AU comparisons and 2017 unit-level SAARs using existing methods. Part 2 included newly derived R-SAARs. Hospital ASP leads were surveyed to assess their team’s response after each part.

**Figure:**
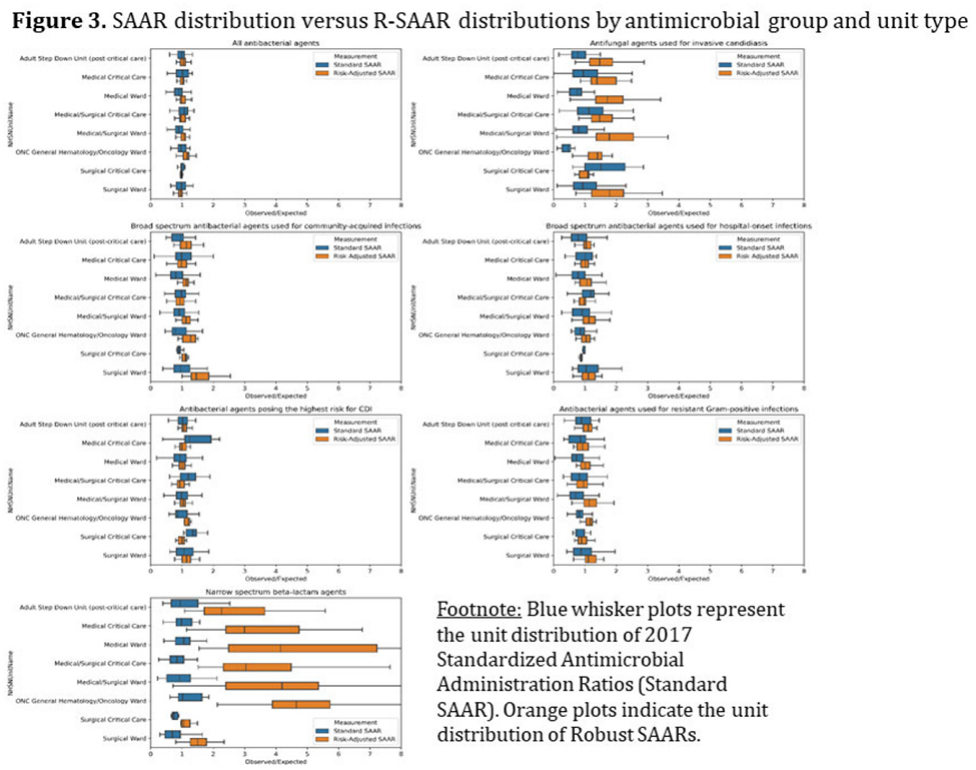

**Figure:**
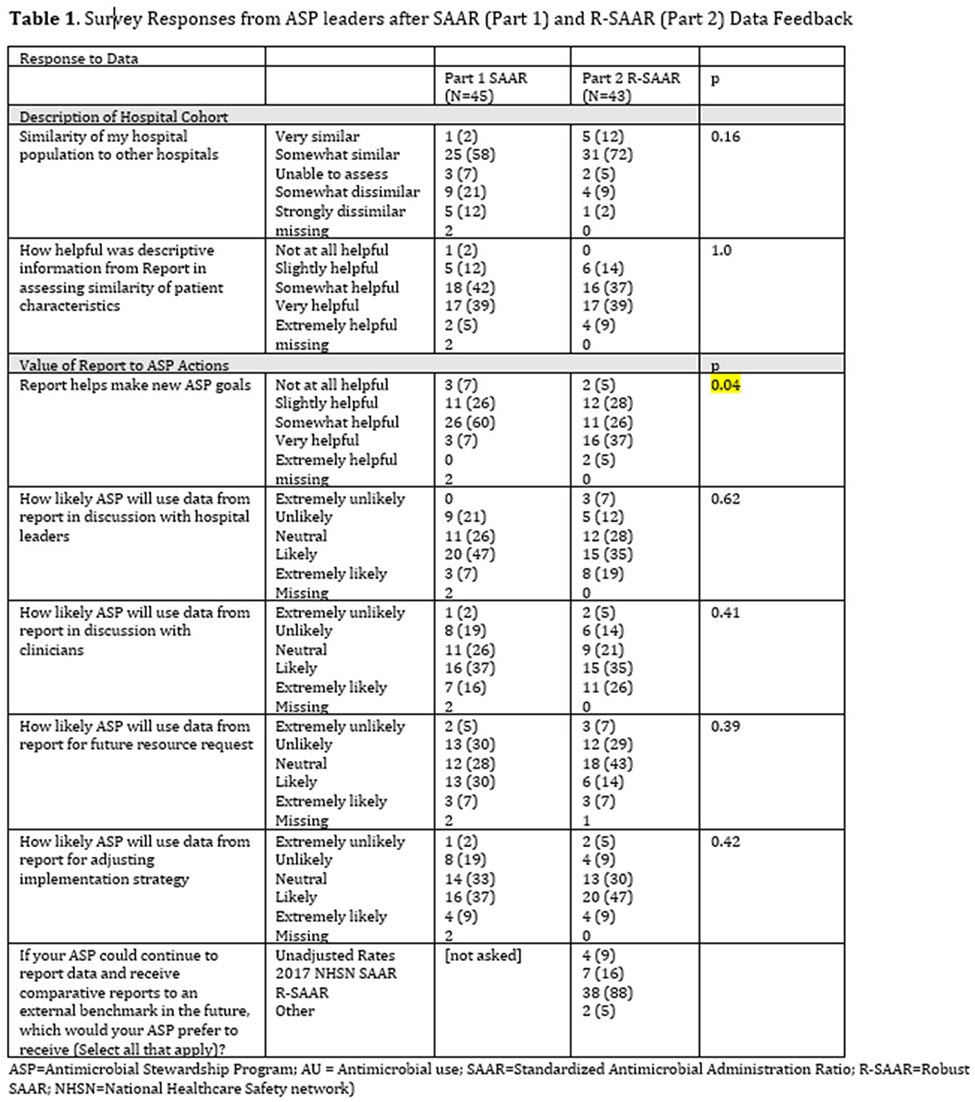

**Results:**

Data included 2.5 million DOT and 5.6 million days present among 50 hospitals in 2022. Unit-level SAARs were calculated for 272 adult and 32 pediatric units. R-SAARs were derived for 474 adult and 45 pediatric units as a larger variety of unit types could be included. Data feedback included hospital- and unit-level O:E values and percentiles (Figures 1 and 2). R-SAARs produced extreme values for antimicrobial groups and units with infrequent AU, but tighter distributions for commonly used groups as compared to SAARs (Figure 3). Forty-five of 50 ASPs voluntarily participated in data feedback; half actively used SAARs at baseline. ASPs perceived Part 2 as being more helpful in developing goals but did not score Part 2 higher in other questions of value (Table 1). Most ASPs (88%) indicated preference for R-SAARs.

**Conclusion:**

When encounter-level risk adjustment was applied, O:E and percentile estimates changed ASP assessments. ASPs preferred encounter-level risk adjustment when asked about desire for future AU comparisons.

**Disclosures:**

Rebekah W. Moehring, MD, MPH, FIDSA, FSHEA, UpToDate, Inc.: Author Royalties Elizabeth Dodds Ashley, PharmD, MHS, HealthtrackRx: Advisor/Consultant|UpToDate, Inc.: Author Royalties Eili Klein, PhD, Beckman-Coulter: Grant/Research Support|Diasorin: Grant/Research Support|MeMed: Grant/Research Support Carlos Santos, MD, MPHS, UpToDate, Inc.: Author Royalties Michael J. Smith, M.D., M.S.C.E, Pfizer: Grant/Research Support

